# Venetoclax: A Novel Treatment for Patients With del(17p) Chronic Lymphocytic Leukemia

**Published:** 2017-09-01

**Authors:** Michelle A. Borg, Amber Clemmons

**Affiliations:** University of Georgia College of Pharmacy and Augusta University Medical Center, Augusta, Georgia

Chronic lymphocytic leukemia (CLL) is a hematologic malignancy characterized by abnormal numbers of mature B lymphocytes, which can accumulate in the bloodstream, as well as lymph nodes and/or bone marrow. Patients may be diagnosed incidentally upon review of routine laboratory testing and do not require treatment until the disease progresses to cause cytopenias and/or symptomatic organ involvement (Packham & Stevenson, 2005).

Chronic lymphocytic leukemia is the most common leukemia diagnosis in the United States, accounting for 25% of the new cases of leukemia in the country (National Cancer Institute, 2016). The American Cancer Society estimates that nearly 20,110 new cases of CLL will be diagnosed, and 4,660 deaths from CLL occur in 2017. The average person’s lifetime risk of developing CLL is about 0.5% (1 in 200), with men having a slightly higher incidence than women (American Cancer Society, 2017). CLL mainly affects older adults, with the average age at diagnosis being 71 years (National Cancer Institute, 2016).

Although approximately 83% of patients are alive 5 years after diagnosis, CLL is not curable with conventional treatment (American Cancer Society, 2017). Early treatment intervention in nonsymptomatic patients who do not have cytopenias has not been shown to be beneficial. Nonsymptomatic patients are followed up based on their level of risk and are counseled to be aware of constitutional symptoms, such as fever, night sweats, and weight loss (Shanafelt, 2006). Once a patient experiences constitutional symptoms, has lymph node involvement, has an enlarged spleen or liver, and/or has anemia or thrombocytopenia, treatment intervention takes precedence. The Rai and Binet staging systems evaluate these factors and aid in prognosis (National Comprehensive Cancer Network, 2016).

Initial treatment of CLL, as recommended by the National Comprehensive Cancer Network (NCCN) Guidelines, is guided by various factors, including the presence or absence of the deletion of 17p. The del(17p), which reflects the loss of the *TP53* gene and can accompany mutations in the remaining *TP53* allele, is associated with worse outcomes, a shorter treatment-free interval, shorter median survival, and poorer response to chemotherapy (NCCN, 2016). Given the poor prognosis in patients with del(17p), there is a need for further research and development of novel drugs in this setting.

Currently, first-line therapy for relapsed or refractory disease in this subset of patients includes ibrutinib (Imbruvica) monotherapy (NCCN, 2016). Venetoclax (Venclexta) is the first drug approved by the US Food and Drug Administration (FDA) for CLL patients who have del(17p) and have received at least one prior therapy.

## MECHANISM OF ACTION AND PHARMACOKINETICS

Venetoclax is a first-in-class, highly selective and orally bioavailable small-molecule inhibitor of B-cell lymphoma 2 (BCL-2), which is an apoptotic protein that can be overexpressed in some CLL patients. Overexpression of BCL-2 has been associated with resistance to chemotherapeutic agents and subsequently poorer outcomes. Venetoclax helps restore the process of apoptosis via multiple mechanisms: direct binding to the BCL-2 protein, displacement of proapoptotic proteins, triggering of mitochondrial outer membrane permeabilization, and activation of caspases (Genentech, 2016).

Venetoclax achieves a maximum plasma concentration within 5 to 8 hours of dosing. The estimated volume of distribution ranges from 226 to 321 L and is highly bound to human plasma protein. Venetoclax is metabolized by the liver predominately via CYP3A4/5. It is cleared via hepatic elimination, with approximately 20% unchanged drug excreted in the feces. The mean elimination half-life is approximately 26 hours, with the pharmacokinetics of venetoclax not changing over time (Genentech, 2016).

## CLINICAL STUDIES

**Phase I Study**

An early phase I dose-escalation study in patients with relapsed or refractory CLL or small lymphocytic lymphoma (SLL), receiving a median of 3 previous therapies (range, 1–11), evaluated venetoclax at 8 dose levels, ranging from 150 to 1,200 mg/day. This study included an expansion cohort in which 60 additional patients were treated with a weekly stepwise ramp up in doses as high as 400 mg/day. The first dose administered to patients was 200 mg followed by a washout period of at least 72 hours. After this period, continuous daily administration was initiated at one of the eight dose levels. Patients continued to receive venetoclax until disease progression or unacceptable toxicity (Roberts et al., 2016).

Results of the study revealed laboratory tumor lysis syndrome (TLS) in all three patients in the first group that received a single initial dose of 200 mg or 100 mg. Subsequent patients were initiated with a test dose of 20 mg or 50 mg and later underwent a ramp up in dose to designated doses of 150 mg, 200 mg, 300 mg, 400 mg, 600 mg, 800 mg, and 1,200 mg if no TLS developed. In an expansion cohort, 60 patients received 400 mg/day after starting 20 mg/day in an extended stepwise ramp up. The most common reasons for treatment discontinuation were progressive disease (35%), toxicity (11%), and eligibility for allogeneic stem cell transplantation (6%). Tumor lysis syndrome occurred in 10 of 56 patients (18%) in the escalation cohort. Overall, neutropenia was the most common grade 3 or 4 adverse event (41%, 48 of 116 patients; Roberts et al., 2016).

The pooled overall response rate (ORR), which was defined as the proportion of patients with decreased tumor burden after treatment, was 77%, with 30% having either a complete response (CR), defined as no evidence of disease after treatment, or a complete response with incomplete count recovery (CRi). Among patients with del(17p) CLL, the response rate was 71%, with 16% CR. The median duration of progression-free survival was 25 months (95% confidence interval [CI]: 17–30 months) for patients in the dose-escalation cohort. The estimated 2-year overall survival for all patients was 84%. Richter’s transformation was more common among patients with del(17p) CLL (56% vs 44%; Roberts et al., 2016).

**Phase II Studies**

A phase II, single-arm, multicenter study was conducted in patients with relapsed or refractory CLL to determine the proportion of patients achieving an overall response with venetoclax monotherapy. The ORR was 79.4% (85 of 107; 95% CI: 70.5%–86.6%) by an independent review committee assessment. Of the 85 responders, 8 (8%) had CR/CRi, 3 (3%) had nodular partial remission, and 74 (69%) had partial remission (Stilgenbauer et al., 2016).

With a median duration of follow-up of 12.1 months (interquartile range [IQR], 10.1–14.2 months), the median time to first response was 0.8 months (IQR, 0.1–1.7 months), and the median time to CR/CRi was 8.2 months (IQR, 6.7–10.0 months). The estimated percent of patients still responding at 12 months by Kaplan-Meier estimate was 85% (95% CI: 75%–91%). Consistent with the phase I study, neutropenia was the most common grade 3 or 4 event, which occurred in 40% of patients (Stilgenbauer et al., 2016).

**Ongoing Research and Future Studies**

Venetoclax received accelerated approval from the FDA on April 11, 2016, for the treatment of patients with CLL and del(17p) who have been treated with at least one prior therapy. Furthermore, the FDA granted venetoclax breakthrough therapy designation, priority review, and orphan drug designation (FDA, 2016). Due to the accelerated approval, further studies are required to evaluate the efficacy and safety of venetoclax.

Currently, there are multiple studies evaluating the use of venetoclax in CLL patients. Of note, there are two phase III studies evaluating venetoclax as monotherapy for CLL patients with del(17p) or *TP53* mutation who have received a prior B-cell receptor inhibitor (ClinicalTrials.gov identifiers NCT02756611 and NCT02980731). Additionally, a variety of current studies are assessing the use of venetoclax with other agents in the treatment of CLL. Several of these trials include the combination of ibrutinib, obinutuzumab (Gazyva), rituximab (Rituxan), and/or bendamustine (ClinicalTrials.gov identifiers NCT02756897 and NCT02758665).

Additional studies are recruiting patients to assess venetoclax in other disease states, including multiple myeloma, myelodysplastic syndromes, non-Hodgkin lymphoma, and follicular lymphoma, primarily in combination with other agents.

## DOSING, ADMINISTRATION, MODIFICATIONS, AND SPECIAL POPULATIONS

At this time, venetoclax is exclusively indicated for patients with CLL with del(17p), as detected by an FDA-approved test, who have received at least one prior therapy. Recommended dosing includes a 5-week ramp-up schedule to the final total daily dose of 400 mg (Table 1). The 5-week ramp-up dosing schedule is designed to gradually reduce tumor burden and decrease the risk of TLS (Genentech, 2016).

**Table 1:**
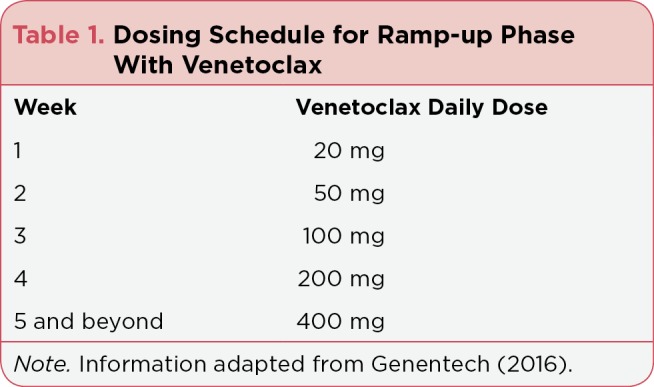
Dosing Schedule for Ramp-up Phase With Venetoclax

Venetoclax should be taken with a meal and water at approximately the same time each day. Tablets should be swallowed whole; therefore, patients should be counseled not to chew, crush, or break open tablets prior to swallowing. A starting pack of venetoclax provides the first 4 weeks of medication according to the ramp-up schedule. Once the ramp-up phase is completed, the 400-mg dose is achieved using four of the 100-mg tablets. If a dose is missed within 8 hours of the time it is usually taken, the missed dose should be taken as soon as possible, and the normal daily dosing schedule should be resumed. If the dose is more than 8 hours late, the missed dose should not be taken, and the normal daily dosing should be resumed the following day. In the event a patient vomits after a dose, no additional dose should be taken that day (Genentech, 2016).

Confirmed by clinical trial data, venetoclax can cause rapid reduction in tumor burden and poses a risk for TLS in the initial 5-week ramp-up phase. Changes in blood chemistries can occur as early as 6 to 8 hours following the first dose of venetoclax and after each dose increase. Thus, TLS prophylaxis and monitoring are required, with specific recommendations stratified based on tumor burden in patients receiving venetoclax (Table 2; Genentech, 2016). Some patients may require inpatient monitoring upon initiation and dose-escalation periods. Additionally, patients may have prolonged visit times in the outpatient setting due to blood chemistries needing to be interpreted in real time to allow for potential same-day interventions. 

**Table 2:**
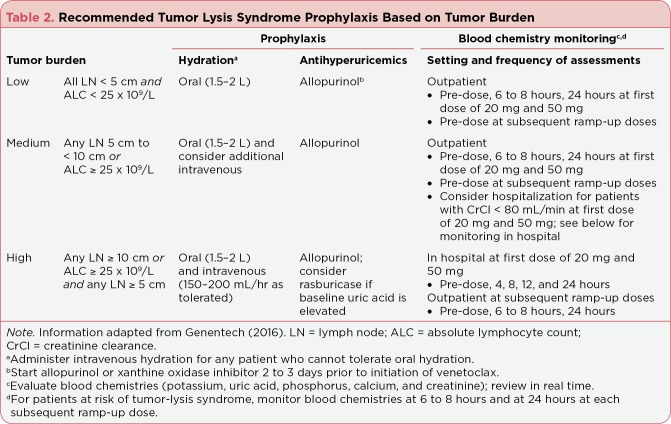
Recommended Tumor Lysis Syndrome Prophylaxis Based on Tumor Burden

Dose modifications are required for patients who experience toxicity and those taking CYP3A and P-glycoprotein (P-gp) inhibitors. Toxicities requiring dose modification include blood chemistry changes or symptoms suggestive of TLS, grade 3 or 4 nonhematologic toxicities, grade 3 or 4 neutropenia with infection or fever, and grade 4 hematologic toxicities (except lymphopenia). First occurrences involve the interruption of venetoclax with no dose modifications. Second and subsequent interruptions require dose modifications (Table 3). Venetoclax discontinuation should be considered in patients who require less than 100 mg for more than 2 weeks.

**Table 3:**
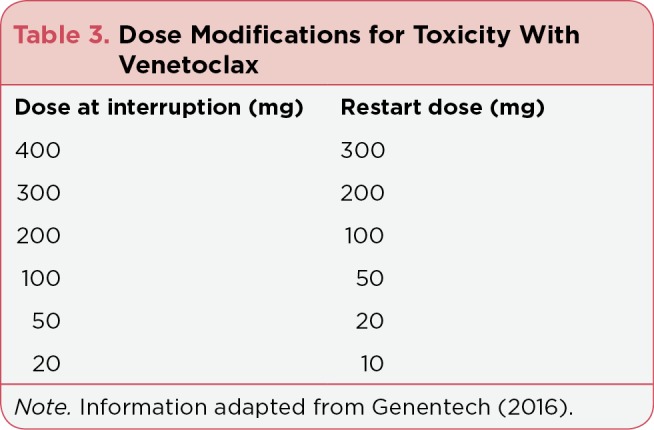
Dose Modifications for Toxicity With Venetoclax

Concomitant use of venetoclax with strong CYP3A inhibitors at initiation and during ramp-up phase is contraindicated due to increased venetoclax exposure and risk of TLS. If venetoclax is at steady daily dose and strong CYP3A inhibitor use is warranted, the venetoclax dose can be reduced by at least 75%. The combination of venetoclax and moderate CYP3A or P-gp inhibitors should be avoided or the dose of venetoclax should be reduced by at least 50% (Genentech, 2016).

## ADVERSE EFFECTS

The primary adverse effects noted in phase I and II trials were neutropenia, anemia, and thrombocytopenia. Other common adverse events (> 10%) included diarrhea, nausea, vomiting, fatigue, pyrexia, and upper respiratory tract infections (Table 4; Roberts et al., 2016; Stilgenbauer et al, 2016). These side effects are similar to those with ibrutinib therapy. Due to the risk of TLS, patients in the phase II clinical trial received prophylaxis and management. This strategy is reflected in the FDA-approved package insert of the medication discussed here.

**Table 4:**
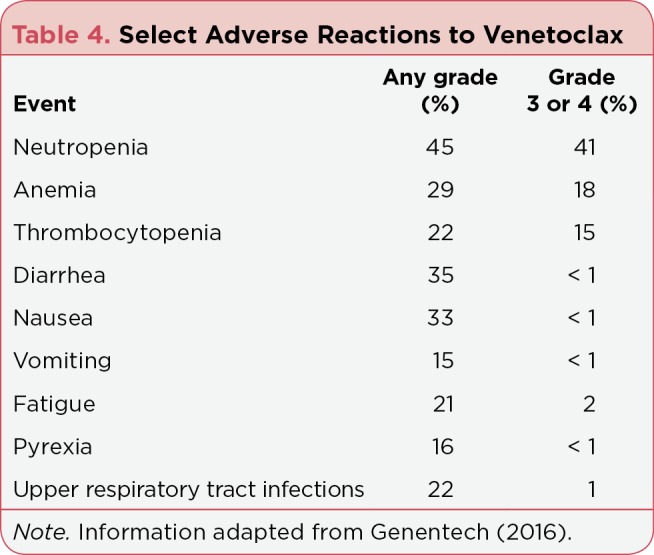
Select Adverse Reactions to Venetoclax

## ROLE OF ADVANCED PRACTITIONERS

Venetoclax is the first FDA-approved drug that targets the BCL-2 protein and is indicated for patients with del(17p) CLL who have received at least one prior therapy. Patients with del(17p) have worse outcomes and poor responses to chemotherapy, thus the need for novel agents in this population is imperative. Compared with ibrutinib, venetoclax has a similar cost and route of administration.

Advanced practitioners (APs) have an opportunity to optimize the management of patients with CLL who are receiving venetoclax. They can provide patient and family counseling, monitor and manage adverse effects, and deliver health-care professional education. With venetoclax, monitoring of blood chemistries is crucial in the management of these patients to prevent TLS. 

Required monitoring recommendations are stratified based on tumor burden and occurs at initiation and each dose titration. Some patients may need to be monitored initially in the inpatient setting, and outpatient visits may be prolonged due to real-time reading of laboratory values. Although these monitoring strategies may be inconvenient for patients, APs have the opportunity to guide hydration and hyperuricemic management as well as frequency of laboratory monitoring, with subsequent interventions for laboratory or clinical TLS if warranted. Due to the initial dosing and monitoring complexity of venetoclax, APs have the ability to help direct transitions of care for patients once the regimen is established and less monitoring is required.

Advanced practitioners can also play an active role in evaluating each patient for clinically relevant drug interactions. Dose adjustments or changes in medication therapies are warranted for concomitant CYP3A and P-gp inhibitors. Venetoclax doses may also need to be adjusted for patients who experience toxicities. Consultation with a clinical pharmacy specialist in hematology/oncology may be useful.

## CONCLUSION

The treatment of patients with del(17p) CLL has remained a challenge, given poor response to conventional therapies. The impressive clinical data of venetoclax, showing overall response rates of 85% and a median time to first response of 0.8 months, make this agent a significant addition to current management strategies. This is reflected in the category 2A designation given to venetoclax in the current NCCN guidelines. Moreover, future studies including venetoclax are diverse and may foreshadow broader utility of this medication.
